# The Role of Neuronal Oscillations in Visual Active Sensing

**DOI:** 10.3389/fnint.2019.00032

**Published:** 2019-07-23

**Authors:** Marcin Leszczynski, Charles E. Schroeder

**Affiliations:** ^1^Department of Neurological Surgery, College of Physicians and Surgeons, Columbia University, New York, NY, United States; ^2^Translational Neuroscience Laboratories, The Nathan S. Kline Institute for Psychiatric Research, Orangeburg, NY, United States

**Keywords:** active sensing, cognition, attention, oscillations, phase reset, eye movement, saccade, predictive coding

## Abstract

Visual perception is most often studied as a “passive” process in which an observer fixates steadily at point in space so that stimuli can be delivered to the system with spatial precision. Analysis of neuronal signals related to vision is generally keyed to stimulus onset, stimulus movement, etc.; i.e., events external to the observer. In natural “active” vision, however, information is systematically acquired by using eye movements including rapid (saccadic) eye movements, as well as smooth ocular pursuit of moving objects and slower drifts. Here we consider the use of alternating saccades and fixations to gather information from a visual scene. The underlying motor sampling plan contains highly reliable information regarding “where” and “when” the eyes will land, this information can be used predictively to modify firing properties of neurons precisely at the time when this “contextual” information is most useful – when a volley of retinal input enters the system at the onset of each fixation. Analyses focusing on neural events leading to and resulting from shifts in fixation, as well as visual events external to the observer, can provide a more complete and mechanistic understanding of visual information processing. Studies thus far suggest that active vision may be a fundamentally different from that process we usually study with more traditional passive viewing paradigms. In this Perspective we note that active saccadic sampling behavior imposes robust temporal patterning on the activity of neuron ensembles and large-scale neural dynamics throughout the brain’s visual pathways whose mechanistic effects on information processing are not yet fully understood. The spatio-temporal sequence of eye movements elicits a succession of temporally predictable quasi-rhythmic sensory inputs, whose encoding is enhanced by entrainment of low frequency oscillations to the rate of eye movements. Review of the pertinent findings underscores the fact that temporal coordination between motor and visual cortices is critical for understanding neural dynamics of active vision and posits that phase entrainment of neuronal oscillations plays a mechanistic role in this process.

## Introduction

**FIGURE 1:**
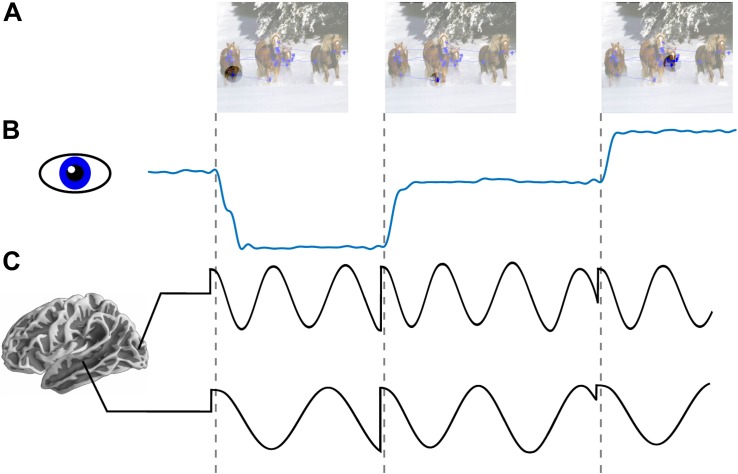
Low frequency phase reset as a mechanism for active sensing. **(A)** An image with overlapped traces from eye tracker showing aggregated eye position across an interval of 1 s (blue line plot). Circle reflects schematic depiction of fovea across three consecutive fixations. **(B)** Horizontal eye position across three saccades. **(C)** Schematic depiction of oscillatory field potential phase reset locked to saccade onset in occipital (alpha ∼8–12 Hz) and medial temporal lobe (theta ∼4–7 Hz). Locally dominant oscillations (i.e., alpha in occipital cortex and theta in MTL) are reset at the time of saccade onset.

In natural vision, information is nearly always available at the retina. Yet, in primates only the central visual field has sufficient density of retinal photoreceptors to permit high-resolution vision (Curcio et al., 1990). Consequently, both human and non-human primates sample visual space actively by systematically shifting eye gaze, using the fovea (i.e., the central portion of the retina) to sample points of interest (Figures 1A,B) rather than holding gaze constant and simply absorbing a continuous inflow of input (Yarbus, 1967; Gilchrist et al., 1997). Studies in rodents actively sampling the environment by sniffing and whisking prompted the use of the term “active sensing” (Ahissar and Arieli, 2001; Szwed et al., 2003; Kleinfeld et al., 2006). Numerous papers have raised the logical proposition, that this active sensing regime should apply to visual sensing in primates including humans (e.g., Ahissar and Arieli, 2001, 2012; Schroeder et al., 2010), and indeed there is evidence for this view (Purpura et al., 2003; Rajkai et al., 2008; Bartlett et al., 2011; Ito et al., 2011; Hoffman et al., 2013; Jutras et al., 2013; Hamamé et al., 2014; Staudigl et al., 2017; Katz et al., 2018; Barczak et al., 2019). Despite these well-known attributes of natural *active* visual behavior, experiments designed to study visual sensory and cognitive functions usually require participants to fixate gaze at a central location while visual stimuli are presented. This traditional “passive” approach, has been extremely productive, but it has several major limitations. First, the very idea that eye position is *ever* static during awake viewing, even when fixation is required, is countered by a wealth of empirical observations (see review by Ahissar and Arieli, 2001). Second, eye movements reflect the observer’s information gathering strategy, and the traditional paradigm disconnects the timing and quality of visual input from this strategy. Finally, because of this disconnection, the ordering of perception and action is obscured; i.e., in natural vision, actions usually precede and follow sensory inputs forming a basic sampling triplet eye movement-perception-eye movement. Because the major sensory input (entering the retina at fixation onset) is in effect “*caused*” by the eye movement, the timing of the associated visual input is largely predictable at the time of movement initiation. Thus, unlike the traditional approach, in natural vision, the timing of sensory input is controlled by the brain’s motor sampling routine and can be utilized in processing of sensory information (Yarbus, 1967; Bahill et al., 1975). In short, there is a fundamental distinction between visual Active Sensing, and the more passive processes studied with gaze held constant.

In this Perspective we highlight the role of active saccadic exploration in visual cognition. In particular, we review evidence supporting a role of predictive phase reset of neuronal oscillations (see Figure 1C) which enhances sensory processing by amplifying responses and by facilitating transfer of information between areas. Next, we consider the idea that this signal, entrained by a motor sampling (saccadic exploration) routine, modulates local neural activity and organizes perception into discrete “events” constraining “sampling rate” of the visual system. Third, we explore the complementarity of our perspective with earlier work on active sensing in vision and somatosensation (Ahissar and Arieli, 2001, 2012). Finally, we outline translational implications of Active Sensing and some future directions.

## A Role for Low Frequency Neural Oscillations in Active Visual Sensing

**FIGURE 2:**
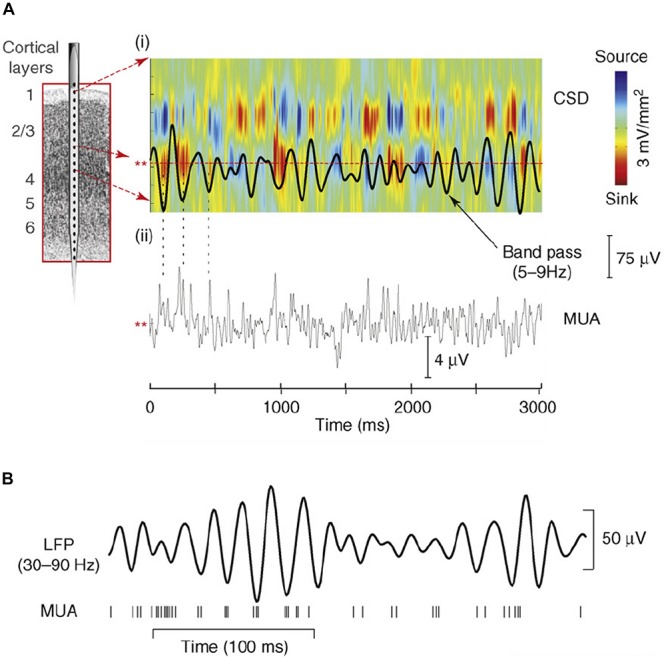
**(A,i)** Theta-band (5–9 Hz band pass) oscillatory activity from a lower supragranular site in primary auditory cortex (asterisks at left) superimposed on the underlying current source density (CSD) profile for the supregranular layers. Net outward transmembrane current flow generates net extracellular current sources (blue), whereas net inward current flow generates current sinks (red). The theta oscillation at this site represents the “underside” of the superficial current dipole so that negative deflections correspond to current sinks and positive deflections reflect current sources, alternating at a theta rhythm. **(A,ii)** Multiunit activity (MUA) simultaneously recorded from the same site. Drop lines are provided to show the relationship between the initial three negative deflections and sinks at this site and MUA correlates. Note that current sinks and sources correspond to MUA peaks and troughs, indicating alternations in local neuronal excitability. **(B)** Relation between gamma-band (30–90 Hz) oscillatory phase and neuronal firing (MUA) from a recording in macaque visual area V4. Vertical lines at the bottom represent occurrence of action potentials. Panel **(A)** reprinted from Schroeder and Lakatos (2009) with permission from Elsevier. Panel **(B)** reprinted from Womelsdorf et al. (2006) with permission from Springer.

Field potentials (FP) in scalp and intracranial recordings are largely generated by synchronous transmembrane currents occurring in ensemble of neurons around the electrode site (Mitzdorf, 1985; Schroeder et al., 1995). Analysis of the current source density (CSD) profiles across layers of primary sensory cortex revealed that oscillations in FP are generated by rhythmic pulses of transmembrane current flow which reflect shifts between depolarized and hyperpolarized states in the local neuronal ensemble (Lakatos et al., 2005; Womelsdorf et al., 2006; Haegens et al., 2015; see Figure 2). Accordingly, low frequency oscillatory phase modulates neural firing (Buzsáki and Draguhn, 2004; Lakatos et al., 2005; Figure 2), as well as the related broad-band high frequency activity (BHA; Ray et al., 2008; Leszczynski et al., 2019) sampled from the pial surface of the cortex (e.g., Canolty et al., 2006; Leszczyński et al., 2017). Because of this, the phase at which visual input arrives will determine whether it is amplified or attenuated (Schroeder and Lakatos, 2009) and consequently the probability that the input will be perceived. Under the Active Visual Sensing hypothesis, the neural circuitry that generates saccades is the source of signals which predictively reset ongoing oscillations to the high-excitability phase at the time of fixation onset.

An accumulation of findings across sensory systems (Ahissar and Arieli, 2001; Szwed et al., 2003; Kleinfeld et al., 2006; Schroeder et al., 2010; Morillon et al., 2015, 2016; Merchant et al., 2015; Tort et al., 2018) and even the motor system (Saleh et al., 2010) suggests that active sensing employs entrainment of euronal excitability fluctuations (oscillations) to facilitate information processing. Entraining neuronal oscillations to the rhythm of the sampling strategy can help the observer to reduce and chunk or otherwise structure sensory input streams into sequences of perceptual events. This has two profound consequences for cognition and neural dynamics. First, because only some information is being sampled, chunking limits or “down-samples” the amount of information that must be processed, thereby minimizing computational burden and optimizing use of resources. Second, quasi-rhythmic sampling behavior (i.e., the spatio-temporal pattern of eye movements) with its deterministic properties allows predictive modulation of neuronal excitability in sensory systems, further optimizing sensory encoding. In the context of visual active sensing, neuronal entrainment of sensory cortices is believed to be orchestrated by outputs from motor system which predictively reset oscillations throughout the system to a high excitability phase near the time of fixation onset (Purpura et al., 2003; Rajkai et al., 2008; Bartlett et al., 2011; Ito et al., 2011; Hoffman et al., 2013; Jutras et al., 2013; Staudigl et al., 2017; Katz et al., 2018; Barczak et al., 2019). By aligning ensemble excitability fluctuations with a rhythmic event stream, entrainment can potentially amplify neuronal responses to the events in the stream (Lakatos et al., 2008); in this case, events correspond to inputs that enter at the moment of fixation (Rajkai et al., 2008). Based on earlier work, phase entrainment to the saccade-fixation-saccade cycle could potentially lower perceptual thresholds (VanRullen, 2016), and provide a reference frame for spike-phase coding (Kayser et al., 2009).

## Local Neural Activity Reflects an Interaction Between Saccade- and Input-Locked Signals

Every time we move our eyes a volley of visual input is initiated in the retina and this information is then processed by a succession of neural ensembles in areas staged along the brain’s visual pathways (Felleman and Van Essen, 1991). Because the retinal input during each eye movement is suppressed (Ross et al., 2001) as early as lateral geniculate nucleus of the thalamus and V1 (Sylvester et al., 2005), information that propagates through the visual hierarchy are temporally structured forming a sequence of “visual samples.” According to the Active Visual Sensing hypothesis as outlined here, local neural response to visual samples reflect an interaction between retinal “driving” inputs and “modulatory” non-retinal signals. The former carry visual information and can cause (drive) action potentials. The latter signals control (modulate) the probability that the retinal inputs will drive action potentials, and they likely originate in the network of cortical and sub-cortical areas involved in saccade planning (for review see Girard and Berthoz, 2005; Gilbert and Li, 2013). The modulatory signals operate in part by phase-reset of ongoing low frequency oscillations (Ahissar and Arieli, 2001; Szwed et al., 2003; Lakatos et al., 2008; Schroeder and Lakatos, 2009). This should be evident as increased inter-trial phase coherence in oscillatory activity at the rate of saccades (see Figure 1C).

Indeed, signals with such characteristics have been observed in several parts of the primate brain including primary visual cortex (Rajkai et al., 2008; Ito et al., 2011) but also higher order cortices like the temporal lobe and superior temporal sulcus (Purpura et al., 2003; Bartlett et al., 2011), as well as medial temporal lobe and the hippocampus in humans (Hoffman et al., 2013; Staudigl et al., 2017; Katz et al., 2018) and non-human (Jutras et al., 2013) primates. Importantly, stronger phase reset has been associated with saccade onset rather than fixation onset (Ito et al., 2011; Katz et al., 2018) which is in line with its “top-down” and “modulatory” as opposed to “bottom-up” and “driving” characteristics. This non-retinal signal seems to operate by interacting with local population activity in producing excitation (Rajkai et al., 2008) and modulating the timing of visually evoked spiking at fixation onset (Ito et al., 2011). Local activity during natural exploration would therefore reflect an interaction between saccade-locked “modulatory” and input-locked “driving” signals.

## Discrete Perception as a Consequence of Quasi-Rhythmic Exploration

According to the Active Sensing hypothesis at each fixation low frequency oscillations are reset to the high-excitability phase modulating neural activity and consequently perceptual threshold. In line with this suggestion attention performance is not uniform across saccade-fixation-saccade cycle but increases close to fixation onset (Jonikaitis et al., 2012; see also Deubel and Schneider, 1996; Rolfs et al., 2011). The current hypothesis suggests further that perceptual threshold should oscillate rhythmically at the rate of exploration with intervals of higher and lower performance (see also VanRullen and Koch, 2003). This hypothesis has gained substantial support particularly in the visual system. For example, the phase of ongoing EEG oscillations in range of alpha/theta was observed to predict near-threshold perception and modulate the likelihood of detecting a target (Busch et al., 2009; see also Mathewson et al., 2009). Similarly, the phase of ongoing oscillations has been observed to influence peri-saccadic mislocalization (McLelland et al., 2016). These effects establish a relation between the phase of ongoing oscillations and perception suggesting that visual inputs are not processed equally across time. Importantly, covert spatial attention, which can shift across a scene independently of eye position, nonetheless shares common control circuits with overt saccadic search (Rizzolatti et al., 1994; Kustov and Robinson, 1996; Corbetta, 1998). Periodicity in visual perception has been shown to originate from rhythmic sampling imposed by attention (Busch and VanRullen, 2010; VanRullen et al., 2014; VanRullen, 2016). This is further supported by work from Fiebelkorn et al. (2013) who sampled near-threshold perception during systematically varied cue-target interval and observed attention cycling at 4–8 Hz (see also Landau and Fries, 2012; Song et al., 2014). This rhythmicity has been also hypothesized to depend on dynamic interplay within the fronto-parietal (“eye movement control”) network in both human (Helfrich et al., 2018) and non-human (Fiebelkorn et al., 2018) primates. Altogether, these studies imply that like perception, attention is periodic rather than continuous with underlying sampling in theta/alpha frequency range.

A blinking or flickering spotlight of attention and perception with repeated fluctuations in perceptual acuity lasting several 100 ms might appear an inefficient mechanism if considered without further assumptions. However, one could argue that such a mechanism is advantageous for survival, as it allows the brain to monitor events outside of the focus of attention (e.g., Landau and Fries, 2012; Fiebelkorn et al., 2013).

## Visual Active Sensing Beyond Exploratory Saccadic Sampling

We use large exploratory saccades which predictively relocate the fovea in a search for information as our prime example of active sensing, because they are easy to measure accurately and because vision in human and non-human primates relies heavily on these movements (Yarbus, 1967; Gilchrist et al., 1997; Liversedge and Findlay, 2000; Henderson, 2017). However, similar principles apply to other types of eye and body movements across sensory modalities. For example, the same active sampling strategy is applied in olfactory and somatosensory processing as exemplified by sniffing (Mainland and Sobel, 2005; Wachowiak, 2011) and whisking (Ahissar and Arieli, 2001; Kleinfeld et al., 2006) in rodents. Indeed the conceptual and empirical foundations for our work come from these studies. On the one hand, several parts of our formulation dovetail with earlier formulations of visual active sensing (Ahissar and Arieli, 2001, 2012), particularly as applied to fixational eye movements (FeyeM) in primates. FeyeM are the very small (<<1.0^∘^) drift, and tremor eye movements that occur during visual fixation (Ahissar and Arieli, 2001). Most have supposed that these, along with “microsaccades” (Martinez-Conde et al., 2013), serve to keep retinal images from fading during fixation, though Ahissar and Arieli (2001, 2012) have proposed that FeyeM in particular reflect an active sensing mechanism that allows fine detail and texture vision at a level of resolution beyond that offered by the density of cone receptors in the retina. They have also speculated that retinal activity driven by FeyeM may drive thalamocortical oscillatory activity loops that help the visual system form a systematic representation of visual form texture across the surface of V1. On the other hand, several of our findings prompt conclusions about visual active sensing that differ distinctly from earlier ones. In particular, activity in the oscillatory thalamocortical loops associated with FeyeM are specifically driven by retinal input, whereas neuronal oscillations tied to larger saccades are ambient dynamics that are predictively phase reset by non-retinal input associated with saccade generation (Rajkai et al., 2008; Barczak et al., 2019). Additionally, we observe that saccades are basically rhythmic or quasi-rhythmic, while that seems less clear with FeyeM. Similarly, in saccades the eyes move conjointly, while this may not be true for FeyeM. Finally, while we would concur on most of the assumptions made by the earlier formulations (Ahissar and Arieli, 2001, 2012), a few (e.g., “processing of the retinal signals evoked immediately after saccades likely involves significant suppression” and “in natural vision there are no strobe flashes”) may be untenable at this point. Regarding the former, it is clear that in natural viewing, V1 neuron excitability is significantly enhanced immediately at saccade offset (Barczak et al., 2019).

There is potential complementarity between saccades and FeyeM that is intriguing. On the one hand, saccades and FeyeM could engage in a basic alternation during natural viewing, with prolonged fixation periods interrupting more rhythmic and rapid saccadic search. On the other hand, with saccadic search operating at 3–4 Hz, and fixations lasting between 200 and 300 ms (e.g., Barczak et al., 2019), there is ample time for drifts (∼10 Hz) and tremors (∼40 Hz) to operate between saccades. This being the case, small rapid FeyeM would be nested within larger slower saccades, much as higher frequency amplitude nests within lower frequency phase in the resting EEG (Lakatos et al., 2005). The proposition that drift-type FeyeM have a tendency to occur at 10 Hz (Ahissar and Arieli, 2012; Herrmann et al., 2017), is also intriguing in light of the finding that in free viewing, phase reset at the rate of saccades is accompanied by phase reset in the higher alpha (8–12 Hz) range. This fits with the idea that some drift FeyeMs are coupled to saccades. Moreover, high amplitude alpha range activity occurs between saccades (Barczak et al., 2019) and alpha oscillations modulate V1 neuronal excitability so strongly that neurons fluctuate between high firing and near zero firing in response to visual input (Haegens et al., 2015). This could in effect produce a “strobe flash-like” modulation that would reduce retinal smearing by FeyeM, as proposed earlier (Ahissar and Arieli, 2012).

Like exploratory saccades, microsaccades have been observed to predictively modulate neural activity and behavior (Rucci et al., 2007; Bosman et al., 2009; Ko et al., 2010; Chen et al., 2015; Bellet et al., 2017; Loughnane et al., 2018; Lowet et al., 2018a, b). For example, using recordings from superior colliculus and frontal eye fields in non-human primates, Chen et al. (2015) observed enhanced neural firing for peripheral stimuli that preceded microsaccades and suppression of neural firing for stimuli presented immediately after microsaccades. This was further supported by research showing that visual behavior in a discrimination task depends on the presence of microsaccade periods with behavioral costs and benefits oscillating relatively to movement onsets (Bellet et al., 2017). These findings suggest that microsaccades are associated with a motor preparatory signal that impacts sensory processing and behavior. Thus, in most respects microsaccades are more like saccades than FeyeM.

As all eye movements in primates involve relocation of the fovea, it is possible that a moving fovea is critical for sensorimotor coupling in vision. Alternatively, sensorimotor coupling in animals with no fovea might rely on other sources of predictive modulation in visual encoding. Is therefore vision of animals with no fovea (e.g., a mouse) less dependent on signals from the motor system? A mouse vision operates at a very low resolution (Prusky and Douglas, 2004) with its entire retina being similar to peripheral retina in primates (Huberman and Niell, 2011). Nevertheless, activity in its primary visual cortex depends on feedback signals from motor cortex and is sensitive to locomotion and mismatch between actual visual information and locomotion-based predictions (Niell and Stryker, 2010; Keller et al., 2012; Saleem et al., 2013; Attinger et al., 2017; Leinweber et al., 2017; for review see Keller and Mrsic-Flogel, 2018; Pakan et al., 2018). Neural oscillations in gamma range and neural firing in mouse V1 are both increased during periods of locomotion as compared to equivalent visual stimulation during stationary intervals (Niell and Stryker, 2010). Importantly, the effect of active (i.e., locomotion-dependent) stimulation has not been observed in the visual thalamus, suggesting it might reflect cortical feedback to V1 rather than changes in peripheral sensory stimulation.

Saccades, albeit demonstrably central to visual information processing in primates, are part of an overall repertoire that includes distinctly different forms of oculomotor behavior like microsaccades, FeyeM, as mentioned above, as well as smooth pursuit (e.g., Lisberger et al., 1987), which were not discussed here. A more detailed review of these movements is beyond the scope of this discussion. It is, however, clear that in all of these cases, sensing depends causally on motor activity (i.e., eye/vibrissal/hand/respiratory movements) sharing the very same assumption that motor output provides information which is fed back to sensory systems where it modulates local neural activity. A complete understanding of Active Sensing needs to include the whole range of eye movements as well as other forms of sensorimotor coupling.

## Translational Implications of Active Sensing

Despite theoretical consequences of the acknowledgment that most of sensory input is preceded and followed by a movement, Active Sensing has also translational implications. Autism, schizophrenia, ADHD are only few examples where entrainment to visual or auditory stimuli is impaired (for review see Calderone et al., 2014; Simon and Wallace, 2016). Because Active Sensing involves entrainment of a large and distributed neural network including multiple cortical and subcortical areas which participate in highly specialized functions (like preparing and executing eye movement, integrating information across saccades, etc.). Neurological impairment to any particular part of this network should manifest itself in highly specific disruptions to the dynamics of active exploration and therefore would be diagnostically useful (Thakkar et al., 2017). For example, 2–6-months-old infants later diagnosed with autism spectrum disorders fixate less on the eyes while viewing natural images of faces (Jones and Klin, 2013). Similarly, the temporal pattern of fixations is altered in children with dyslexia even before they develop any reading disability (Benfatto et al., 2016). The visual search pattern is impaired in patients suffering from Alzheimer disease (Molitor et al., 2015) and mild cognitive impairment (Lagun et al., 2011). The Parkinson patients tend to fixate longer while visually exploring complex images. Importantly, the magnitude of this effect scales with disease progression (Archibald et al., 2013). All these findings suggest that active sampling behavior is both selectively and specifically impaired in neurodevelopmental disorders. This does not assume that these disorders are caused by an intrinsic deficit in visual or oculomotor system but rather that they manifest its early onsets in these systems and alter visual sampling behavior.

## Future Directions

The studies described above suggest that the active visual exploration elicits neural dynamics in low frequencies (i.e., theta, alpha) similar to multiple cognitive processes like attention (Schroeder and Lakatos, 2009; Saalmann et al., 2012), memory encoding and retrieval (Sederberg et al., 2003; Osipova et al., 2006; Lega et al., 2012), working memory (Jensen and Tesche, 2002; Jokisch and Jensen, 2007; Sauseng et al., 2010; Leszczyński et al., 2015, 2017) as well as cognitive control (for review see Cavanagh and Frank, 2014). Because stimulus complexity, task and subjects’ goal all strongly modulate viewing parameters (Yarbus, 1967; see Voss et al., 2017 for recent review), it remains a possibility that changes in low-frequency neural dynamics currently attributed to particular cognitive functions are confounded with changes in spatio-temporal patterns of active visual exploration. Therefore, future studies rather than attempting to minimize the amount of eye movements should include them as a source of relevant information and study neural dynamics in the context of active sensing strategies adopted by participants. For example, it remains an open question how memory encoding and retrieval depends on individual sampling strategy and how each of these influence neural oscillations (but see Jutras and Buffalo, 2010; Jutras et al., 2013; Staudigl et al., 2017, 2018; see also Voss et al., 2017). In fact, recognition of the critical role of the motor sampling routine in natural active vision raises a host of additional questions such as: (1) How much information is encoded in an individual fixation and how is information accumulated across saccades? (2) Does the phase perturbation observed in a distributed network including early sensory areas as well as the medial temporal lobe and the hippocampus play a direct role in that process? (3) Is the frequency, and duration of phase perturbation constant or does it change from lower to higher visual areas, to medial temporal lobe? (4) What is the role of these distinct nodes during natural exploration; for example, does the low frequency phase reset observed in medial temporal lobe contribute to planning and preparation of the upcoming eye movement? (5) Given that the rate of eye movements changes depending on both external (e.g., scene properties) and internal (e.g., goals) factors, do the parameters of large-scale neural dynamics depend on these changes; for example, do longer fixations lead to phase concentration in lower frequencies which could in turn lead to longer excitability intervals for representing more detailed information encoded by larger ensembles? (6) Related to the last point, do FeyeM and saccades play complementary roles in vision, if not in the larger sense of analyzing “what” vs. “where” information (Ahissar and Arieli, 2001), at least in the sense that FeyeM expand the information gleaned by the saccadic search routines in which they are embedded.

## Summary

Visual perception is often studied as “passive” process in which an observer fixates steadily at a point in space and information is delivered to the system at times arbitrary defined by an experimenter. In natural “active” vision, however, information is systematically acquired by a motor-sampling plan, programming a sequence of alternating fixations and saccades. This sampling behavior imposes a robust temporal patterning on information flow in the visual pathways. While it is widely acknowledged that visual activity throughout the cortical processing hierarchy depends on the interaction between stimulus qualities and properties of neurons in each cortical area, accumulating evidence suggests that during natural free viewing low frequency neuronal oscillations yoked to eye movements predictively modulate neuronal excitability in order to both amplify the neural representation of visual inputs and enhance their transmission through the visual pathways. This “saccadic” version of the active sensing hypothesis builds on and complements earlier formulations based on patterns of whisking and sniffing in rodents and on smaller, more rapid “fixational” eye movements in primates. The active sensing hypothesis acknowledges that the dynamics of eye movements highly depends on task and stimulus properties and that the pattern and rate of saccades will differ across memory loads, stimuli, and overall task demands. The hypothesis also acknowledges that condition-dependent changes to neural dynamics as typically observed in cognition tasks performed during fixation might in fact parallel those associated with overt sampling behavior (e.g., saccade rate, fixation duration, etc.). In any case, the bulk of the evidence we have reviewed points to the conclusion that visual processing in the context of unconstrained eye movements is fundamentally different from that observed in subjects constrained to fixate steadily during visual stimulation.

## Author Contributions

Both authors contributed to the writing and editing of this manuscript.

## Conflict of Interest Statement

The authors declare that the research was conducted in the absence of any commercial or financial relationships that could be construed as a potential conflict of interest.
